# Chyle Leak following Open Donor Nephrectomy: A Rare Complication—A Case Report

**DOI:** 10.1155/2012/259838

**Published:** 2012-12-20

**Authors:** Sandeep Harkar, Dharam Vir Singh, Sanjay Kumar Gupta, Raghav Talwar, Yajvender Pratap Singh Rana

**Affiliations:** Department of Urology, Army Hospital Research & Referral, Delhi Cantt, New Delhi 110012, India

## Abstract

*Introduction*. Donor workup in renal transplantation is extensive. Despite this, chyle leakage following donor nephrectomy, a rare complication, has been reported in the literature. We encountered two cases of chyle leak in kidney donors in our series of open donor nephrectomies. *Summary of Cases*. After complete workup, standard open retroperitoneal donor nephrectomy with drain placement was performed in 684 living renal donors. We encountered chyle leak in two cases. The first case was a 33-year-old female who underwent an otherwise uneventful left donor nephrectomy but continued to have high drain output (upto 300–400 mL/24 hrs) in the postoperative period. The drain fluid was milky, raising the suspicion of chyle which was confirmed on biochemical analysis. The second case was a 42-year-old female with a similar case history. Both were managed conservatively with low-fat diet. The leak subsided spontaneously in three weeks and one week in the first and second patients, respectively. The drain was removed, and the patients remained symptom-free on followup. *Conclusions*. Both of our cases of chyle leak following open donor nephrectomy were managed successfully with conservative management. The management options and the experience of other centers are reviewed and discussed.

## 1. Introduction

Donor workup in renal transplantation is extensive with the aim of ensuring donor safety at all costs and at the same time providing an adequately functioning kidney to the recipient. However, complications may occur. One of them, although rare, is postoperative chyle leak. 

We reviewed our experience of 684 living renal donors since 1991 to date. They had undergone standard open retroperitoneal donor nephrectomy after complete preoperative workup. At the time of closure, a drain was placed in the renal fossa which was removed when the drainage became less than 50 mL/day. We encountered chyle leak in two cases.

## 2. Case Reports

### 2.1. Case 1

**Figure 1:**
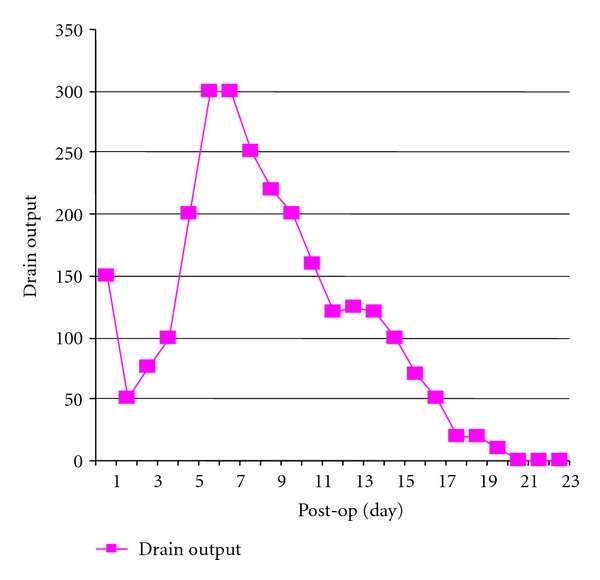
Daily postoperative drain output of Patient number 1.

A 33-year-old female, resident of nonfilaria endemic area, was a voluntary kidney donor for her husband. She underwent an otherwise uneventful left retroperitoneal donor nephrectomy. After an initial fall, drain output started to increase and became milky after postoperative day 3, after commencement of oral feeds (Figure 1). Suspicion of chyle was confirmed on biochemical analysis. (triglycerides: 580 mg/dL (serum TG: 150 mg/dL), TLC: 9200/cumm; lymphocytes: 85%). Drain fluid culture was sterile. Ultrasound abdomen was done which revealed no retroperitoneal lymphadenopathy/collection. She continued to have high drain output—200–300 mL/day for one week.

**Figure 2:**
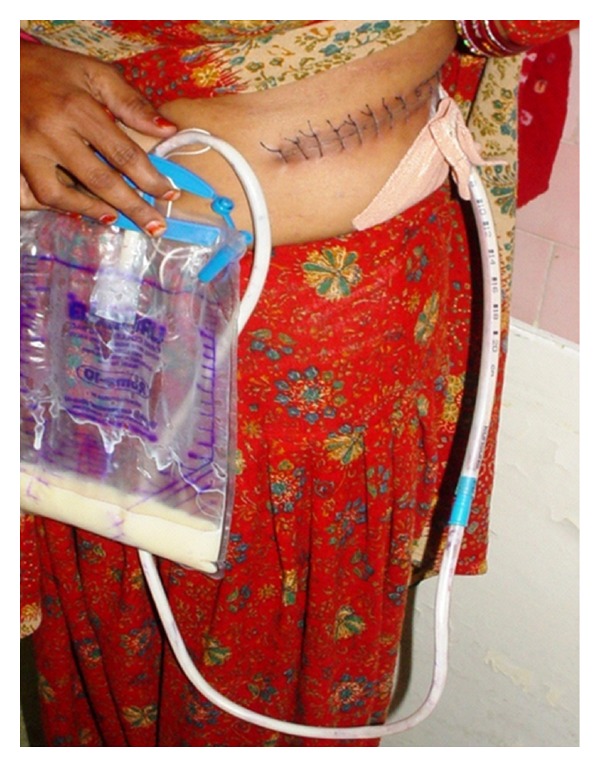
Case number 1 with retroperitoneal drain in situ.

She was managed aggressively and was started on TPN for a week. Drain output reduced, and subsequently oral feeds were restarted comprising of high-protein, low-fat (mainly medium-chain triglyceride) diet. Leak gradually subsided spontaneously in total of three weeks, and at that time the drain was removed (Figure 2). 

### 2.2. Case 2

**Figure 3:**
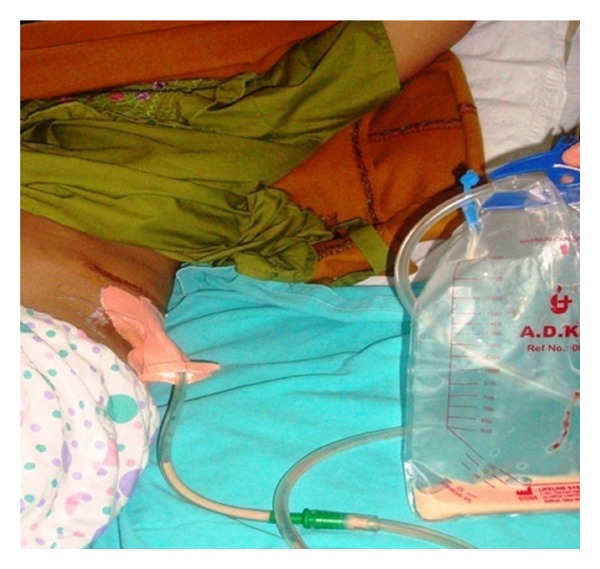
Case number 2 with retroperitoneal drain in situ.

A 42-year-old female was with a similar case history as the first case. She also had undergone left open donor nephrectomy with an uneventful intraoperative period. She had milky fluid in drain after start of oral diet (max output: (200/day)). This patient was also managed conservatively with dietary management. Leak gradually subsided spontaneously in 7 days (Figure 3).

Both patients remained symptom-free on followup. Incidentally, the recipients of both kidneys did well. 

## 3. Discussion

After reviewing the literature for this complication, we found that we are first to report two cases of chyle leak following open donor nephrectomy. However, there are few reports of chylous ascites following laparoscopic donor nephrectomy (Table 1) [1].

Most reports listed the complication occurring after left laparoscopic donor nephrectomy. In our study also, both the cases had undergone left donor nephrectomy. The cause for this finding is not known. It may be incidental as the frequency of left donor nephrectomy is much greater than right donor nephrectomy or this complication is unique to left side. Further inquiries and investigative efforts are needed in this regard.

Chyle leak has been reported after other abdominal surgeries also. Abdominal surgical procedures usually causing chyle leak include Abdominal aortic aneurysm repair (80% of all postsurgical chylous ascites), Retro-peritoneal lymph node dissection, Inferior vena caval resection & Liver transplant [2].

The cause of chyle leak following donor nephrectomy is not exactly known. Chyle leak has been increasingly reported in the recent past, indicating the possible role of inadequate control of perivascular lymphatics during nephrectomy performed by minimally invasive techniques where energy sources like harmonic, LigaSure, and electrocautery are used. These modalities may not be as effective in control of lymphatics as suture ligation or clipping as suggested by Nishizawa et al. with respect to chylous ascites following laparoscopic radical nephrectomy [3].

Treatment options in patients with postoperative chylous ascites primarily involve conservative management in the form of dietary modifications. Constant loss of protein and lymphocytes may cause nutritional and immunological disturbances. The major bulk of fat in diet should comprise of free fatty acids and medium chain triglycerides as these are absorbed directly from the gut into the portal venous circulation. Total parenteral nutrition (TPN) is required in cases who do not respond to dietary modifications. Essential fatty acids and fat soluble vitamins need to be supplemented. Somatostatin has been shown to be effective in one study [4]. Other options in refractory cases include exploration with ligation/clipping of disrupted lymph vessels, application of fibrin glue, povidone-iodine instillation, or placement of peritoneovenous shunt [5, 6].

## 4. Conclusion

Chyle leak following donor nephrectomy may be prevented by meticulous surgical dissection and clipping/ligating all the lymphatic tissue around the renal vessels. It can be managed with conservative treatment in most of the cases.

## Figures and Tables

**Table 1:** 

Author	Year published/laparoscopic (LDN) or open donor nephrectomy (ODN)	Pts (*n*)/case report	Number of Pts with chylous ascites/leak in drain
Shafizadeh et al. [7]	2002/LDN	Case report	1
Molina et al. [8]	2003/LDN	Case report	1
Geary et al. [9]	2004/LDN	Case report	1
Leventhal et al. [10]	2004/LDN	500	2
Wu et al. [11]	2004/LDN	10	1
Ramani et al. [12]	2005/LDN	79	1
Caumartin et al. [13]	2005/LDN	Case report	1
Sharma et al. [14]	2005/LDN	Case report	1
Breda et al., 2006 [15]	2006/LDN	300	2
Bachmann et al. [16]	2008/LDN	164	3
Wadstrom J. [17]	2005/hand-assisted LDN	75	1
Aerts et al., 2009 [1]	2009/LDN	1054	3
